# False Belief Reasoning in Adults with and without Autistic Spectrum Disorder: Similarities and Differences

**DOI:** 10.3389/fpsyg.2018.00183

**Published:** 2018-02-16

**Authors:** Monika Sommer, Katrin Döhnel, Irina Jarvers, Lore Blaas, Manuela Singer, Victoria Nöth, Tobias Schuwerk, Rainer Rupprecht

**Affiliations:** ^1^Department of Psychiatry and Psychotherapy, University of Regensburg, Regensburg, Germany; ^2^Department of Psychology, Ludwig-Maximilians-University, Munich, Germany

**Keywords:** autism spectrum disorder, adults, false belief reasoning, theory of mind, mentalizing

## Abstract

A central diagnostic criteria for autism spectrum disorder (ASD) is the qualitative impairment in reciprocal social interaction and a prominent hypotheses that tried to explain this impairment is the Theory of Mind (ToM) deficit hypotheses. On a behavioral level the critical test for having a ToM, the understanding of false beliefs (FB), is often used for testing ToM abilities in individuals with ASD. Investigating the neural underpinnings several neuroimaging studies revealed a network of areas involved in FB reasoning in neurotypical individuals. For ASD individuals the neural correlates of false belief processing are largely unknown. Using functional magnetic resonance imaging and an adapted unexpected transfer task, that makes it possible to distinguish between the computation of diverging beliefs and the selection of a belief-associated response, we investigated a group of adult high-functioning individuals with ASD (*N* = 15) and an age and IQ matched group of neurotypical adults (NT; *N* = 15). On the behavioral level we found no group differences. On the neural level, results were two-fold: In the story phase, in which participants had to compute whether the character's belief is congruent or incongruent to their own belief, there were no differences between neurotypical participants and those diagnosed with ASD. But, in the subsequent question phase, participants with ASD showed increased activity in the bilateral anterior prefrontal cortex, the left posterior frontal cortex, the left superior temporal gyrus, and the left temporoparietal area. These results suggest that during the story phase in which the participants processed observable actions the neural correlates do not differ between adult individuals with ASD and NT individuals. But in the question phase in which participants had to infer an unobservable mental state results revealed neural differences between the two groups. Possibly, these subtle neural processing differences may contribute to the fact that adult ASD individuals are able to master explicit false belief tasks but fail to apply their strategies during everyday social interaction.

## Introduction

The ability to attribute mental states such as beliefs, intentions, desires, and emotions to oneself and other people is necessary to navigate successfully through the social world and is known as Theory of Mind (ToM) or mentalizing. The understanding of false beliefs is commonly considered to be the critical test for having a ToM. False belief attribution requires a decoupling between a person's mental representation of the world and the real state of the world and enables a person to understand that mental states can misrepresent reality. A classical task for testing false belief understanding is the so-called *unexpected transfer task*, in which a character (e.g., Maxi) leaves an object (chocolate) in one location (e.g., the drawer) and while he or she is outside the room the object is transferred to a new location (Wimmer and Perner, 1983). As a consequence Maxi's subsequent search for the chocolate in the drawer will be unsuccessfully.

In autism, a central diagnostic criteria is a qualitative impairment in reciprocal social interaction. A prominent hypotheses that tried to explain these impairments is the Theory of Mind deficit hypotheses that is based on the observation that individuals with autism spectrum disorders (ASD) show severe deficits in the understanding that in some situations other people have beliefs and other mental states that differ from their own (Baron-Cohen et al., 1985; Senju et al., 2009). As opposed to young neurotypical (NT) children who begin to master false belief tasks at the age of four or five, it is not until the mental age of 6 years that children with ASD pass these tasks (Happé and Frith, 2014). However, from around 12 years of age individuals with ASD and average IQ often show levels of false belief performance that are similar to those of NT children (Happé, 1995). Nevertheless, it seems that adolescents and adults with high-functioning autism or Asperger syndrome fail to employ their knowledge about false beliefs during naturalistic interactions (Ponnet et al., 2004).

Neuroimaging studies that investigated belief reasoning gathered evidence that in healthy adults the posterior part of the medial prefrontal cortex (pMPFC, also referred to as dorsal MPFC), the bilateral temporo-parietal junction (TPJ), the posterior superior temporal sulcus (pSTS) and the precuneus are involved in false belief processing (Saxe and Kanwisher, 2003; Saxe and Powell, 2006; Sommer et al., 2007, 2010; Aichhorn et al., 2009; Scholz et al., 2009; Van Overwalle, 2009; Young et al., 2010; Rothmayr et al., 2011; van der Meer et al., 2011; Döhnel et al., 2012, 2016; Schurz et al., 2013; van Veluw and Chance, 2013; Schuwerk et al., 2014).

There are also some studies that tried to shed light on the neural underpinnings associated with ToM deficits in individuals with ASD. These studies used a variety of different tasks that are supposed to elicit mental state representation resulting in heterogeneous findings (Happé et al., 1996; Castelli et al., 2002; Mason et al., 2008; Kana et al., 2009; Lombardo et al., 2011; Dufour et al., 2013). For example, Kana et al. (2009) investigated adults with autism and control participants during the viewing of animated geometrical shapes. In one condition the movements of the shapes entail the attribution of mental states like thoughts and feelings to the figures. On the behavioral level, neither reaction time nor error rates differed between the two groups. While results revealed no activity differences in posterior ToM regions, like the right pSTS, the ASD group showed lower activation in frontal regions associated with ToM, like the left superior medial frontal gyrus, the left anterior paracingulate cortex, the bilateral anterior cingulate cortex and the left inferior orbitofrontal cortex. Additionally, during the attribution of mental states to animated figures ASD participants compared to controls showed reduced functional connectivity between frontal and posterior (temporal, parietal and occipital) regions and between temporal and occipital areas. The authors suggested that ASD associated difficulties in mentalizing may be the result of a decreased communication and coordination between frontal and other regions of the brain (Kana et al., 2009).

Using a completely different task–making reflective mentalizing or physical judgements about oneself or another person—Lombardo et al. (2011) found that especially the activity of the right temporo-parietal junction (RTPJ) differentiates between adult participants with ASD and control subjects. Behavioral data did not show any group effects, indicating that ASD individuals responded similar to controls while making mentalizing and physical judgements. But, while in controls RTPJ was selectively more responsive to mentalizing than physical judgements, this selectivity was not apparent in ASD individuals. Additionally, individuals with ASD who were more socially impaired had RTPJ responses that were least selective for mental state information, while those who were least socially impaired had RTPJ responses that were relatively more selective for mental state information. Therefore, the authors suggested that the RTPJ is one of the key areas behind the deficits of ASD participants in mental state attribution (Lombardo et al., 2011).

The neural systems that specifically underlie the ability to attribute false beliefs in individuals with ASD were only investigated by Dufour et al. (2013). They compared adult high-functioning individuals with ASD with neurotypical individuals by using verbal stories. In the belief condition the stories described a character who acquired a false belief. These stories were compared to a photo condition in which a physical representation became false, such as an outdated photograph of a map. Behavioral data were not reported, but the fMRI results revealed no differences between neurotypical and autistic individuals, neither in a whole brain analysis nor in a ROI analysis that focused on typical belief-associated areas. The authors suggest that in adults the social cognitive impairments of ASD individuals can occur without differences in activation patterns during the processing of an explicit ToM task.

In sum, results concerning the neural system that underlies ToM deficits in ASD individuals are very heterogeneous. Some authors proposed a lower activity of frontal areas and a decreased functional connectivity between frontal and other areas in ASD individuals as key mechanisms for ToM deficits (Mason et al., 2008; Kana et al., 2009). Other authors emphasized on the role of the RTPJ in the ToM impairment of ASD individuals (Mason et al., 2008; Lombardo et al., 2011). And still others found no activity differences in the brain between ASD and healthy individuals during processing false beliefs (Dufour et al., 2013).

The aim of the current study was to investigate the neurocognitive correlates of false belief reasoning in adult ASD individuals in more detail. In a previous study with healthy adults Schuwerk et al. (2014) adapted the classical unexpected transfer task in order to separate two processing phases: the computation of beliefs and the inference and selection of another's or one's own belief. In neurotypical adults the initial computation phase was associated with activity in the bilateral temporoparietal cortex, the posterior MPFC and the left inferior frontal gyrus (IFG). In the subsequent question phase, conditions in which participants had to consider the other's belief compared to conditions in which they had to respond according to their own belief were associated with activity in the right temporoparietal cortex. Additionally, the authors show that when incongruent beliefs had to be computed activity of the pMPFC inhibited the temporoparietal cortex. These results support suggestions concerning the role of the pMPFC in stimulus-independent processing. Stimulus-independent processing is necessary when the belief of another person becomes false and does not longer correspond to reality (Sommer et al., 2007; Döhnel et al., 2012), e.g., a person's belief that an object is in location A, but meanwhile the object has been transferred to location B. It seems that in these conditions the pMPFC inhibits stimulus-bound processing which helps to process another person's false belief decoupled from one's own perception of reality.

Interestingly, a recent study of the anatomy of white matter networks revealed that ASD individuals compared to NT individuals showed white matter differences in brain areas associated with belief reasoning. White matter differences in ASD were localized to major association and commissural tracts of the frontal lobe (Catani et al., 2016). These tracts connect frontal lobe to more posterior areas of the parietal, limbic and temporal lobe. The results are also in line with functional imaging studies that investigated ToM abilities in individuals with ASD and pointed to a lower degree of synchronization (functional underconnectivity) between MPFC and temporoparietal areas during ToM tasks in adults with ASD compared to neurotypical adults (Mason et al., 2008; Kana et al., 2009; Murdaugh et al., 2012). Interestingly, these areas were also associated with the processing of divergent beliefs in the study by Schuwerk et al. (2014). Therefore, we suggest that the adapted version of the false belief task might be a useful tool to reveal possible differences in the neural correlates associated with false belief processing between individuals with ASD and neurotypical adults. Several behavioral studies have shown, that adults with high-functioning or Asperger autism tend to be just as efficient in understanding explicit false belief tasks as control subjects (e.g., Happé, 1995; Scheeren et al., 2013). With respect to these results we expect no behavioral differences between the two groups. According to our former fMRI study with NT individuals (Schuwerk et al., 2014) we hypothesized that in the NT group the computation of divergent beliefs is related to activity in the temporoparietal cortex and the medial prefrontal cortex. But with respect to studies showing underconnectivity between frontal and temporoparietal areas (e.g., Murdaugh et al., 2012; Catani et al., 2016), we hypothesized that the ASD group will show lower activity in the frontal lobe and in the temporoparietal area. For the question phase we expect higher activity of the right temporoparietal cortex in the NT group compared to the ASD group.

## Materials and methods

### Participants

Fifteen adults with ASD (10 men, mean age = 28.2) and 15 neurotypical (NT) participants (10 men, mean age = 29.87) with no reported history of psychiatric or neurological disorders were included in the study. The ASD participants were recruited through the autism outpatient clinic of the Bezirksklinikum Regensburg, Germany. All participants were diagnosed by specialized psychiatrists and psychotherapists according to the ICD-10 criteria for Asperger syndrome (F84.5, *N* = 11) and autism without intellectual disability (F84.0, *N* = 4) and were tested with a battery of tests including the Wechsler Adults Intelligence Scale-revised (WAIS-R), the Adults Asperger Assessment (AAA, Baron-Cohen et al., 2005; German version Poustka, 2006), Fragebogen zur Sozialen Kommunikation (FSK; the German version of the SCQ; Bölte and Poustka, 2006) the Faux Pas Recognition Test (Baron-Cohen et al., 1999) and the Reading the Mind in the Eyes test (Baron-Cohen et al., 1997).

The groups were matched according to gender, age and IQ measured with the German verbal test Mehrfachwahl-Wortschatz-Test (MWT; Lehrl, 1989) and the non-verbal Grundintelligenztest (CFT 20; Weiß, 1998; see Table 1 for details). All of the participants gave written informed consent prior to their participation and received payment for participating. The study was approved by the local ethics committee of the University Medical Center Regensburg.

**Table 1:** Demographic characteristics.

	**ASD group (*N* = 15)**		**NT group (*N* = 15)**			
	**M**	**SD**	**M**	***SD***	***t*-value**	***p*-value**
Age	28.2	10.4	29.9	12.2	0.40	0.69
Verbal IQ (MWT)	113.5	10.1	112.8	13.9	0.17	0.87
Non-verbal IQ (CFT 20)	122.3	8.7	125.2	11.0	0.81	0.43

*M, means; SD, standard deviation; ASD, autism spectrum disorder; NT, neurotypical*.

### Task

**Figure 1:**
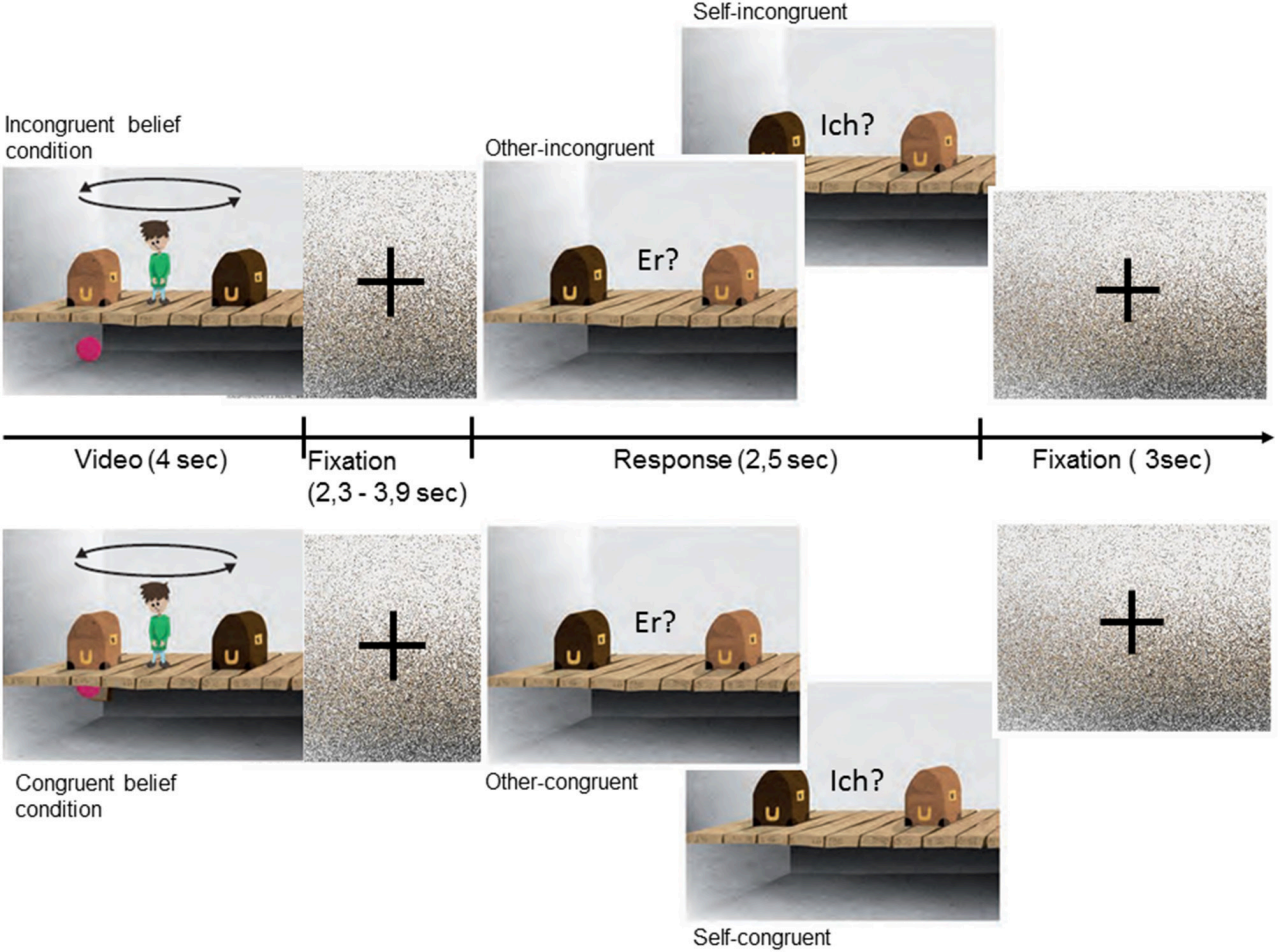
Experimental design: Each trial started with the presentation of a video, in which the protagonist compared to the participant either ended up with an incongruent belief (**Top**) or a congruent belief (**Bottom**) about the location of the ball. The story phase was followed by a fixation picture of scrambled pixels with jittered duration. In the following question phase the participants had to respond either according to their own belief (“Ich?”) or according to the belief of the protagonist (“Er?”) about the ball's location. Response was given via button press. The trial ended with a second fixation picture.

The present task was a version of the object transfer false belief paradigm (Baron-Cohen et al., 1985), adapted from a prior fMRI-paradigm (Schuwerk et al., 2014). Each Trial consisted of two phases (see also Figure 1). In the initial story phase participants watched a 4 s long animation depicted a room with a boy flanked by a dark-brown and light-brown box, which are standing on a wooden floor. Underneath the floor there was an empty basement. In the beginning of each trial a self-propelled-moving ball fell in one of the two open boxes. Then the boxes closed and two events simultaneously happened: the ball bounced into the basement through a hidden trap door, what not could be seen by the story character, and the boxes switched their places, witnessed by the story character. In the congruent-beliefs condition the ball fell into the basement but bounced right back into the same box and was transferred inside the box to the character's other side. In this condition the story character and the participant's belief about the location of the ball were congruent. In the incongruent-beliefs condition, the ball fell into the basement, however, bounced back with a short time delay and entered the other box after they had switched. In this condition the story character's belief and the participant's belief are incongruent. The character believes that the ball was transferred inside the box it initially entered, the participant knows that the ball was not transferred and is now in the other box.

After the initial story phase a fixation picture of scrambled pixels was presented for a varying time interval (jitter) in order to control for possible overlapping BOLD signal responses to stimuli (2.3–3.9 s). Then in the following question phase a still frame was presented for 2.5 s. On this picture the character was replaced and a written test question was presented. Either participants were asked in which one of the two boxes the story character thinks the ball is located (in German: “Er?,” in English: “He?”) or in which box the participant thinks the ball is located (in German: “Ich?,” in English: “I?”). Depending on the prior condition, reasoning about the character's false belief (other-incongruent) or true belief (other-congruent) or reasoning about one's own belief, either diverging from the character's belief (self-incongruent) or corresponding with it (self-congruent), was required. The task of the participants was to respond as fast and accurately as possible. Reaction time (RT) was measured from picture onset until one of two buttons of a five-button fMRI-compatible response pad (LUNItouch, Photon Control Inc., Burnaby, Canada) was pressed. Between trial a fixation picture of scrambled pixels was presented for 3 s.

The animations were controlled for type, number, order, and laterality (ball enters the left or right box first) of events. Question pictures were identical across conditions, showing only the two closed boxes without any social stimuli. After being given a standardized instruction and some practice trials outside the scanner, subjects completed 120 trials, 30 per condition, in a pseudo-randomized order. Presentation software was used for stimulus presentation and response recording (Neurobehavioral Systems Inc., Albany, CA).

### Statistical analysis of the behavioral data

In order to analyze reaction times (RT) and response accuracy (percentage of correct responses) a repeated measures analysis (ANOVA) with within-subject factors of perspective (other vs. self) and congruency of beliefs (incongruent vs. congruent) and the between-subject factor group (ASD, Control) was performed. The significance level for the analyses was set at *P* ≤ 0.05.

### Image acquisition

A 3-Tesla Siemens Allegra Head Scanner (Siemens Inc., Erlangen, Germany) located at the University Medical Center Regensburg was used to record the imaging data. The scanner acquired echo-planar-imaging (EPI) sequences using fast gradients. During T2^*^ data acquisition, we recorded 32 axial slice in interleaved order with a Time-to-Repeat (TR) of 2.0 s, a Time-to-Echo (TE) of 0.95 s, a flip angle of 90°, a Field of View (FoV) of 192 x 192 mm and a voxel size of 3 × 3 × 3 mm. A total of 767 functional images were recorded in the entire experiment.

A structural image was recorded from every subject at the end of functional data acquisition. These T1-weighted images were obtained using a MPRAGE (Magnetization Prepared Rapid Acquisition Gradient Echo) pulse sequence (TR = 2.25 s, TE = 0.026 s, TI = 0.9 s, FoV = 256 mm), scanning 160 slices with voxel size of 1 × 1 × 1 mm^3^.

The entire scan session lasted approximately 35 min.

### Functional imaging data

All images were analyzed with SPM8 (Wellcome Department of Imaging Neuroscience, London, UL) run in Matlab 7.0 (Math Works Inc., Nattick, MA).

Individual subjects' data were slice-timed corrected using the middle slice as a reference. Images were spatially realigned to the first volume by rigid body transformation to correct for head movements. After coregistration to the tructural ${\text{T}}_{1}^{*}$ –weighted images, data were normalized to the functional template contained in SPM8 (Montreal Neurological Institute template, MNI) with a voxel size of 2 × 2 × 2 mm^3^ and smoothed with a 8 mm full-width at half maximum Gaussian kernel.

All statistical analyses were based on functional activity obtained from the whole brain. In the first level analysis, a fixed-effects analysis was computed for each participant based on the general linear model (GLM). The analyses focused on amplitude changes in the hemodynamic response function (HRF) associated with the different mentalizing conditions. For each condition, correctly answered trials were modeled as a boxcar function convolved with the HRF. In the story phase, the two regressors for the incongruent-belief and the congruent-belief condition comprised the last 2 s of the 4 s long video. The events in the video were timed so that exactly after 2 s it became clear whether the story character's belief was congruent or incongruent to that of the participant. In the question phase, which started with the onset of the question and lasted for 2.5 s a regressor for each of the four conditions of interest (other-incongruent, other-congruent, self-incongruent, self-congruent) was modeled. In addition to the regressors of interest, the realignment parameters, the mean constant over scans, and a non-hit parameter (incorrect responses and misses) were included as regressors of no interest. The data were high-pass filtered with a frequency cutoff at 128 s.

Statistical parametric maps (SPMs) were generated for each subject by t-statistics derived from contrasts utilizing the HRF (Friston et al., 2002). To identify brain activity associated with the processing of incongruent vs. congruent beliefs in the story phase, we contrasted the two conditions incongruent-beliefs vs. congruent beliefs.

In order to detect brain activity associated with divergent beliefs in the question phase, we analyzed the main effect of congruency of beliefs [(other-incongruent + self-incongruent) vs. (other-congruent + self-congruent)]. Additionally, the main effect considered person was calculated [(other-incongruent + other-congruent) vs. (self-incongruent + self-congruent)]. Further we analyzed the interaction effect between the considered person and congruency of beliefs [(other-incongruent–other-congruent) > (self-incongruent–self-congruent)] and vice versa.

For group analyses single-subjects' first-level contrasts were introduced in second-level random-effect analysis. First, one-sample *t*-tests for all contrasts were conducted separately for ASD and controls. Second, in order to test the influence of autism we investigated the interaction between group (ASD and Controls) and condition in the story phase (congruency) as well as in the question phase (congruency, perspective). Additionally, we explored whether common brain regions are associated with processing incongruent beliefs in the story phase and in the question phase. Separately for the NT and the ASD group, we computed a conjunction analysis (based on the Minimum statistic compared to the Null Conjunction; Nichols et al., 2005) on the contrasts (story phase: incongruent > congruent beliefs) and [question phase: (self-incongruent + other-incongruent) > (self-congruent + other-congruent)]. The resulting set of significant voxel values for each contrast constituted SPM maps that were thresholded at *p* < 0.001 (uncorrected, 10 or more contiguous voxels). Reported significant voxels survived a statistical FWE (family-wise error)-corrected threshold of *p* < 0.001 for multiple comparisons on cluster level. The activated brain regions were overlaid on the MNI template and labeled according to the Talairach atlas (http://www.bioimagesuite.org/Mni2Tal).

## Results

### Behavioral results

Regarding accuracy, the ANOVA revealed a significant main effect for congruency of beliefs [*F*_(1, 28)_ = 11.90, *p* < 0.01]. Participants gave more correct answers when they were asked for congruent beliefs (*M* = 97.44, *SD* = 3.30) compared to incongruent beliefs (*M* = 93.67, *SD* = 5.61). There was no influence of group for congruency [congruency × group: *F*_(1, 28)_ = 0.09, *p* = 0.76]. For perspective neither the main effect [*F*_(1, 28)_ = 0.09, *p* = 0.78] nor the interaction perspective × group[*F*_(1, 28)_ = 1.07, *p* = 0.31] were significant. Also the interaction congruency × perspective [*F*_(1, 28)_ = 0.15, *p* = 0.7] was not significant. Only the interaction congruency × perspective × group interaction [*F*_(1, 28)_ = 4.64, *p* < 0.05] shows a significant effect.

The analysis of RT revealed a similar picture. Only the main effect congruency [*F*_(1, 28)_ = 136.74, *p* < 0.001] was significant. RT were slower when incongruent beliefs had to be processed (*M* = 1366.10 ms, *SD* = 249.96 ms), compared to processing congruent beliefs (*M* = 977.67 ms, *SD* = 194.49 ms). There were no significant effects for the interaction congruency x group[*F*_(1, 28)_ = 0.02, *p* = 0.89], the main effect perspective [*F*_(1, 29)_ = 0.02, *p* = 0.88], the interaction perspective x group [*F*_(1, 28)_ = 1.62, *p* = 0.21], the interaction congruency x perspective [*F*_(1, 28)_ = 0.66, *p* = 0.42], the interaction congruency x perspective x group [*F*_(1, 28)_ = 1.03, *p* = 0.32]. Altogether behavioral data show no group differences and no influence of the considered perspective. For both groups mean RT and response accuracy for each experimental condition are shown in Table 2.

**Table 2:** Mean reaction time and response accuracy.

	**ASD group**		**NT group**	
	**RT (ms)**	**PCR (%)**	**RT (ms)**	**PCR (%)**
	**M (SD)**	**M (SD)**	**M (SD)**	**M (SD)**
Other-incongruent	1410.1 (258.1)	91.6 (7.2)	1308.2 (270.0)	95.3 (6.9)
Other-congruent	1053.1 (189.8)	97.1 (3.5)	911.7 (195.9)	97.8 (3.9)
Self-incongruent	1418.6 (233.0)	94.0 (5.7)	1328.9 (279.2)	93.8 (5.9)
Self-congruent	1007.1 (169.9)	96.7 (3.1)	938.5 (221.1)	98.2 (4.5)

*RT, reaction time; PCR, percentage of correct responses; M, means; SD, standard deviation; ASD, autism spectrum disorder; NT, neurotypical*.

### Brain imaging results

#### Story phase

**Figure 2:**
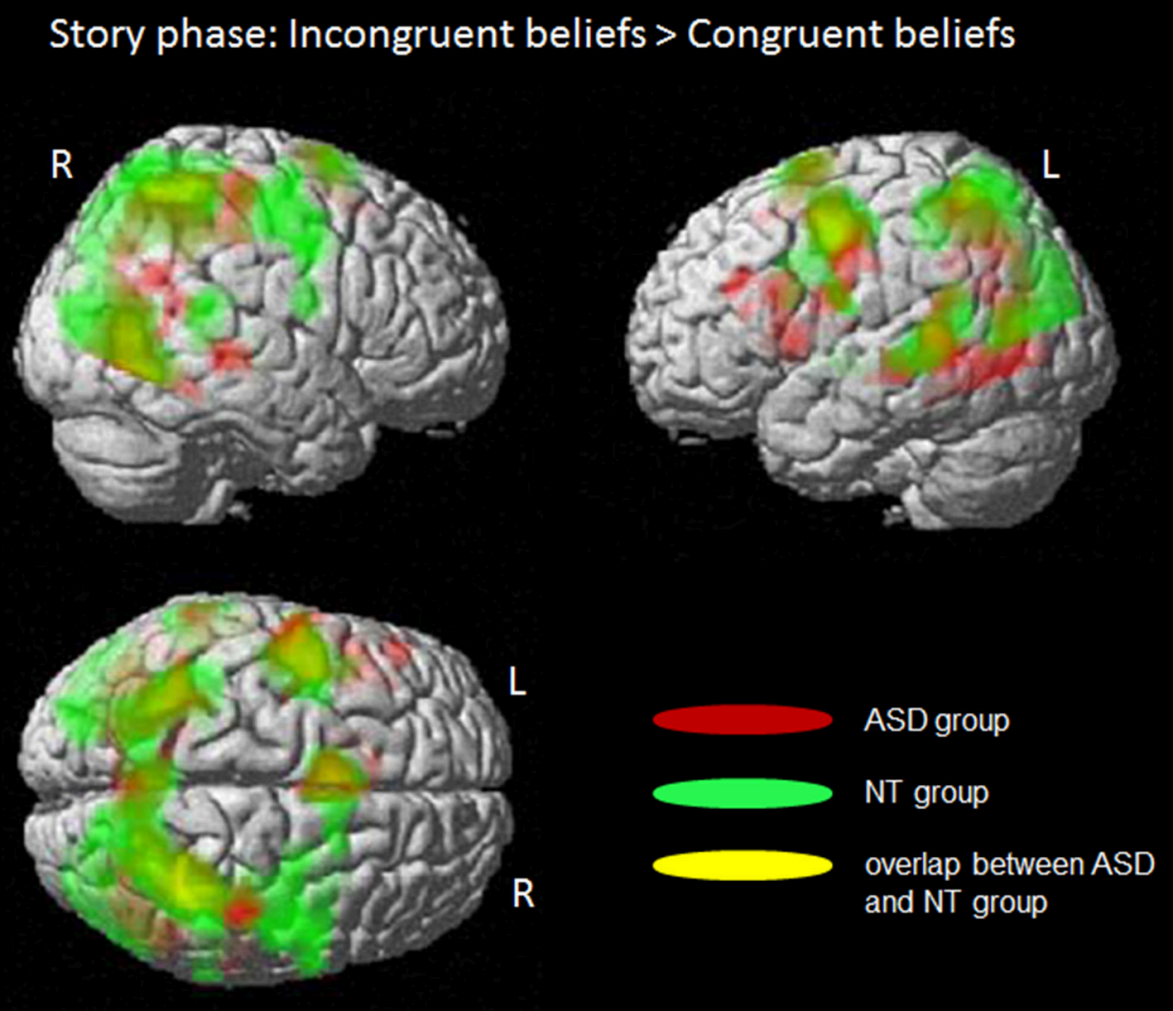
Story phase: Processing of incongruent beliefs compared to congruent beliefs. Whole brain fMRI findings for the ASD group (in red) and the NT group (in green). Common activity in both groups is shown in yellow; L, left side; R, right side. Colored regions indicate significantly activated voxels with *T* > 5.0, P_FWE−corr_ ≤ 0.001, cluster level. There were no significant group differences.

In the story phase, for the participants it became clear whether the character's belief is congruent or incongruent to their own belief. However, they did not know whether they have to respond in respect to their own or the characters' belief. The analyses of this phase focused on brain regions engaged in the processing of emerging incongruent beliefs by contrasting incongruent vs. congruent beliefs (see Figure 2 and Table 3 for more details).

**Table 3:** Whole brain imaging results for the neurotypical (NT) group, the autism spectrum (ASD) group and the whole group: Significant clusters (P_FWE-corr_ ≤ 0.001, cluster level) of functional activity: **(A)** Story phase: peak activation associated with the processing of incongruent beliefs, **(B)** Question phase: peak activation associated with the selection of incongruent and congruent beliefs, **(C)** peak activation associated with group differences during the processing of the question phase, and **(D)** conjunction of story and question phase: peak activation commonly associated with the processing of incongruent beliefs.

		**MNI coordinates**				
**Contrast / Brain region**	**BA**	***x***	***y***	***z***	**Cluster size^a^**	***T*-value^b^**
**(A) Story phase: incongruent > congruent beliefs**						
**NT GROUP**						
Posterior medial prefrontal cortex (pMPFC)	6	8	8	66	795	7.60
Left posterior/inferior frontal gyrus (IFG)	6/44	−40	0	42	1,680	14.91
Left middle temporal gyrus (MTG)	22	−64	−32	2	1,069	7.21
Right middle temporal gyrus (MTG)	22	50	−40	14	530	6.18
Right inferior temporal gyrus	19/39	48	−58	−8	12,712	11.59
Left Thalamus		−8	−28	−2	316	4.19
**ASD GROUP**						
Posterior medial prefrontal gyrus (pMPFC)	6	−8	6	66	515	4.66
Left posterior/inferior frontal gyrus (IFG)	6/44	−50	−10	42	1000	7.00
Left inferior frontal gyrus (IFG)	44	−42	18	24	504	4.44
Right inferior temporal gyrus	19/37	42	−66	−6	7,789	9.78
**WHOLE GROUP**						
Posterior medial prefrontal cortex (pMPFC)	6	−6	6	66	429	10.08
Left posterior/inferior frontal gyrus (IFG)	6/44	−46	−4	44	727	10.03
Right posterior/inferior frontal gyrus (IFG)	6/4	50	−2	34	253	5.45
Left middle temporal gyrus (MTG)	22	−60	−26	0	535	7.79
Right middle temporal gyrus (MTG)	22	52	−38	16	96	6.60
Left inferior temporal gyrus	19/7	−40	−68	8	1,308	10.05
Right inferior temporal gyrus	37/19	46	−60	−8	3,058	11.60
Left Thalamus		−8	−28	−2	28	6.47
**(B) Question phase: Incongruent (self-incongruent + other incongruent) > congruent (self-congruent + other congruent)**						
**NT GROUP**						
Right inferior frontal gyrus^c^ (IFG)	47	42	20	−6	208	4.84
Right inferior temporal gyrus^d^	19	42	−44	2	289	5.53
**ASD GROUP**						
Posterior medial prefrontal gyrus^e^ (pMPFC)	6	−4	6	64	238	5.44
Right inferior frontal gyrus^f^ (IFG)	44	32	28	10	258	5.12
**Question phase: Congruent (self-congruent + other congruent) > incongruent (self-incongruent + other incongruent)**						
**NT GROUP**						
Anterior medial prefrontal cortex (aMPFC)	10/11	14	40	−14	882	6.03
Right posterior frontal gyrus	8	26	24	54	263	6.03
Left posterior frontal gyrus	8	−20	26	54	280	5.67
Right temporoparietal area	40	68	−24	26	416	7.11
Precuneus	31	12	−56	26	526	5.01
**ASD GROUP**						
Anterior medial prefrontal cortex (aMPFC)	10	−6	54	−10	2109	9.61
**(C) Question phase: group differences**						
**NT > ASD**						
Middle occipital gyrus	19/18	−12	80	18	4,103	9.24
		14	−56	−2		7.81
**ASD > NT**						
Left anterior prefrontal gyrus	10/11	−22	52	0	2,119	7.53
Right anterior prefrontal gyrus	10/11	38	48	−12	904	6.94
Left posterior frontal gyrus	6/8	−42	4	60	629	6.89
Left superior temporal gyrus	41/21	−40	−30	8	498	6.23
Left temporoparietal area	39/40	−58	−46	36	612	5.58
**(D) Conjunction of story and question phase: (incongruent>congruent beliefs) and [(self-incongruent + other-incongruent) > (self-congruent + other-congruent)]**						
**ASD GROUP**						
Posterior medial prefrontal cortex (pMPFC)^c^	6	−4	8	64	295	5.42

*Brodmann areas (BAs) are approximate*.

a *Number of activated voxels per cluster*.

b *Peak T-value in activated cluster*.

c *P_FWE−corr_ = 0.021, cluster-level*.

d *P_FWE−corr_ = 0.005, cluster-level*.

e *P_FWE−corr_ = 0.015, cluster-level*.

f *P_FWE−corr_ = 0.011, cluster-level*.

The full factorial ANOVA design with the two factors “group” (ASD/NT) and “congruency” (incongruent/congruent) revealed no significant main effect for “group” or a significant interaction between “group and congruency.” But there was a significant main effect “congruency.” Further t-contrasts revealed that the processing of incongruent compared to congruent beliefs induced activity in a network of brain areas, including the posterior medial prefrontal cortex (BA6), the bilateral posterior/inferior frontal cortex (BA 6/44), the bilateral middle temporal gyrus (BA 22), the bilateral inferior temporal gyrus (BA 19/7/37) and the left thalamus.

The analyses of the separated groups revealed rather similar results. In the control group of NT participants the processing of incongruent beliefs in contrast to congruent beliefs were associated with activity in the posterior medial prefrontal cortex (BA 6), the left posterior/inferior frontal gyrus (BA 6/44), the bilateral middle temporal gyrus (BA 22), the right inferior temporal gyrus (BA 19/40) and the left thalamus.

In the ASD group incongruent beliefs compared to congruent beliefs induced more activity in posterior medial prefrontal gyrus (BA 6), the middle and left posterior/inferior frontal gyrus (BA 6/44), the left inferior frontal gyrus (BA 44) and the right inferior temporal gyrus (BA 19/37).

In both groups, for the reverse contrast, congruent-beliefs condition over incongruent-beliefs condition, no brain area showed significantly increased activity.

#### Question phase

**Figure 3:**
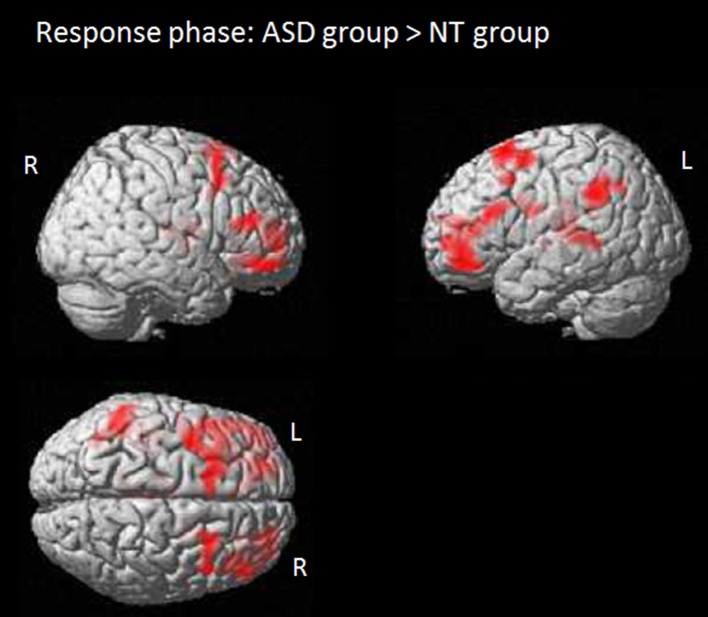
Response phase: activation maps for the contrast ASD group > NT group. Colored regions indicate significantly activated voxels with *T* > 5.0, P_FWE−corr_ ≤ 0.001, cluster level.

The question phase focused on identifying brain regions related to processing a response conflict due to diverging mental states. Results based on the full factorial design with the factors perspective (self/other), congruency (congruent/incongruent) and group (ASD/NT) revealed no main effect for perspective, the interaction perspective x group, the interaction perspective x congruency or the interaction congruency x group. However, there was a main effect for congruency associated with activity in the anterior MPFC/anterior cingulum (BA 10/32). Further analysis revealed that the activity was induced by responses to congruent compared to incongruent beliefs and was independent of group. Results also showed a main effect of group. The comparison between the two groups revealed that in the NT group compared to the ASD group the processing of the response was associated with more activity in the middle occipital gyrus (BA 19/17/18). ASD individuals showed more activity during the question phase in several areas, including the bilateral anterior prefrontal cortex (BA 10/11), the left posterior frontal gyrus (BA 6/8), the thalamus, the left superior temporal gyrus (BA 41/21) and the left temporoparietal area (BA 39/40; see also Figure 3). There were no brain regions in which NT compared to ASD individuals showed significantly increased activity.

We then examined the functional activity within each of the two groups. In the control group, the comparison between the other-condition in which the participants had to consider the other's belief with the self-condition in which the subjects had to respond according to their own belief showed no significant activity. However, independently of perspective, incongruent compared to congruent beliefs were associated with activity in the right inferior fontal gyrus (BA 47) and the right inferior temporal gyrus (BA 19). Congruent beliefs compared to incongruent beliefs induced more activation in the anterior MPFC (BA 10/11), the bilateral posterior frontal gyrus (BA 8), the right temporoparietal area (BA40) and the precuneus.

Also in the ASD group there was no influence of perspective (self, other). But in individuals with ASD the response to congruent beliefs compared to the response to incongruent beliefs induced more functional activity in the anterior MPFC (BA10). There were no significant effect for the reverse contrast incongruent > congruent beliefs (for more details see Table 3).

#### Common activity in the story and question phase

The whole brain conjunction analysis of the contrasts (story phase: incongruent > congruent beliefs) and [question phase: (self-incongruent + other-incongruent) > (self-congruent + other-congruent)] showed only for the ASD group on a lower cutoff for FEW-corrected results (P_FWE-corr_ = 0.021, cluster level) commonly increased functional activity of the posterior medial prefrontal cortex (BA 6) associated with the processing of incongruent beliefs in both the story and the question phase. For the NT group the conjunction analysis revealed no common functional activity.

## Discussion

Autistic symptoms in adult individuals are highly heterogenous and vary considerably with many different aspects like clinical severity, psychiatric impairments, language abilities and intellectual abilities (Howlin et al., 2004). However, in everyday situations deficits in understanding other person's behavior on the basis of their mental states are present in all individuals with ASD and represent a significant barrier to social integration (Howlin et al., 2004; Frith and de Vignemont, 2005). This observation is in line with experimental behavioral studies showing that children and adults with high-functioning or Asperger autism are able to master explicit ToM tasks (Ponnet et al., 2004; Scheeren et al., 2013), but that they show subtle ToM impairments in more complex mentalizing tasks (Begeer et al., 2012; Backer van Ommeren et al., 2015).

Studies that investigated the neural underpinnings of mentalizing in ASD individuals revealed a rather heterogeneous picture. Whereas, some results pointed to lower activity of frontal areas and a decreased functional connectivity between frontal and other areas (Mason et al., 2008; Kana et al., 2009) or to decreased activity of the RTPJ during the processing of ToM tasks (Mason et al., 2008; Lombardo et al., 2011) in ASD individuals, other studies found no activity differences in the brain between ASD and healthy individuals during mentalizing (Dufour et al., 2013). These studies used a variety of tasks that require very different mentalizing abilities, such as the attribution of mental states to moving geometrical shapes or making judgements about another person on the basis of descriptions.

The aim of the present study was to investigate the neural correlate of a very basic ability necessary for mentalizing, the false belief understanding, in adults with and without ASD. We know from several neuroimaging studies that in neurotypical individuals the posterior MPFC, the bilateral TPJ, the middle temporal gyrus and the precuneus are involved in false belief processing (Sommer et al., 2007; Meinhardt et al., 2011; Rothmayr et al., 2011; Döhnel et al., 2012). And we know from ToM studies with ASD individuals that there might be an anatomic and functional underconnectivity between two brain areas that are centrally involved in false belief processing, the MPFC and the temporo-parietal junction (Mason et al., 2008; Kana et al., 2009; Murdaugh et al., 2012).

In order to investigate, if the possible underconnectivity between MPFC and temporo-parietal areas might play a role in false belief processing in individuals with ASD, we used an adapted version of the classical unexpected transfer task (Schuwerk et al., 2014) that enables a separation between the computation of diverging beliefs and the consideration of another's or one's own belief.

As expected, the behavioral results of the present study revealed no differences between the two groups. The ASD as well as the NT adults performed very well with over 90% of right answers in all conditions. This result is in line with former behavioral studies showing that individuals with high-functioning or Asperger autism are able to compensate mentalizing deficits in explicit false belief tasks (Happé, 1995; Scheeren et al., 2013).

### Story phase: computing diverging beliefs

In contrast to our hypothesis, the fMRI results for the story phase, in which participants had to compute divergent beliefs, showed no differences in brain activity between neurotypical participants and those diagnosed with ASD. In both groups the processing of incongruent beliefs was associated with activity in the pMPFC, the left inferior frontal gyrus and the right inferior temporal gyrus. The activation pattern of the whole group is very similar to that of our previous study (Schuwerk et al., 2014) and is also in line with findings from earlier studies on false belief reasoning (Sommer et al., 2007; Rothmayr et al., 2011; van der Meer et al., 2011; Döhnel et al., 2012; van Veluw and Chance, 2013). In the story phase participants had to compute if reality (and therefore their own belief) becomes discrepant from the belief of the story protagonist. The involvement of the pMPFC especially in this phase of the task indicates that the region plays a central role in the processing of incongruent beliefs (Schuwerk et al., 2014). Activity of the bilateral middle temporal gyrus was also associated with computing another's discrepant belief. The region seems to play an important role in the processing of mental states that can be deduced from perceived actions (for an overview see Beauchamp, 2015). In the story phase of the present task it was very important to track the movement of the ball (did the ball jump back into the boxes before the boxes switched or did it jump in the other box after the position change of the boxes?). Based on the ball's movement, the participants had to build their own internal mental model. In the incongruent beliefs condition the movement of the ball led to diverging mental models (the own belief in contrast to the other's false belief), in the congruent condition there were no differences between the mental model of the participant and the mental model of the story character.

### Question phase: considering and selecting own or another's belief

In the question phase, in order to give a correct response participants had to select the previously encoded own or other's belief. Neither for the ASD nor for the NT group an effect of perspective was found. This result contradicts our previous study in which Schuwerk et al. (2014) found increased functional brain activity in the temporoparietal junction for conditions in which the other's belief had to be considered in contrast to conditions in which subjects were asked for their own belief. In contrast, in the present study we found differences in the functional activity rather associated with the processing of congruency than of perspective. These diverging results may be associated with the necessary adaptations of the used paradigm. Schuwerk et al. (2014) used another fixation picture (a picture of the two boxes) and intermixed filler trials with experimental trials in order to prevent habituation. For ASD individuals the scanning procedure with the noise and the narrowness of the scanner is very stressing therefore we reduced the scanning time by skipping the filler trials.

Also results revealed no significant interaction between group and congruency. On a more liberal threshold, in both groups responses to incongruent beliefs compared to congruent beliefs were associated with activity in the right inferior frontal gyrus. Activity of the IFC has been observed in many ToM studies (Mar, 2011) and was primarily found in the response phase of a task (Samson et al., 2005; Sommer et al., 2007; Aichhorn et al., 2009; Döhnel et al., 2012). As a basic process, the right IFG has been observed to be associated with response inhibition (Aron, 2007; Aron et al., 2014). Consistent with this view, the IFC is involved in false belief tasks putting high vs. low demands on self-perspective inhibition (Samson et al., 2005; van der Meer et al., 2011). During false belief reasoning, in order to correctly indicate the location where the protagonist will search for an object, participants have to inhibit their own knowledge about the object's location. By contrast, in a true belief task, the participant's perspective and the perspective of the story character are the same. With respect to the current study, it is argued that the highest demands for response control were required when participants had to inhibit their self-perspective on the ball's location in the incongruent belief condition, in which their knowledge about the ball's location did not match with the knowledge of the story character.

Responses to congruent beliefs compared to responses to incongruent beliefs induced in both groups increased activity in the anterior MPFC. This finding is consistent with Döhnel et al. (2012) and Sommer et al. (2007) who also observed aMPFC activity for the contrast true over false belief reasoning. The aMPFC does play an important role in ToM (Carrington and Bailey, 2009; Frank, 2011). Additionally, the area is also discussed to be a core region of the brain's default network, that is centrally involved in processing self-referential thoughts (Raichle, 2015). Meta-analysis reported an overlap in aMPFC activity associated with ToM processing and the default network (Schilbach et al., 2008; Spreng and Grady, 2009). In our congruent belief condition participants have to respond according to their own knowledge about reality, this might have resulted in activity of the aMPFC, an area which is centrally involved in self-referential thoughts.

## Conclusion

In contrast to our hypothesis that group differences would be observable in the story phase, results revealed significant differences between individuals with ASD and neurotypical individuals only in the question phase. These activity differences were independent of perspective or congruency. During the question phase the ASD group compared to the NT group showed increased activity in the bilateral anterior prefrontal gyrus, the left posterior frontal gyrus, the left superior temporal gyrus and the left temporoparietal area. The anterior prefrontal gyrus is associated with episodic retrieval and the integration of diverse information content (Reynolds et al., 2006), a process generally necessary in contextualizing stimuli and planning (Koechlin et al., 1999; Koechlin and Summerfield, 2007). And the superior temporal gyrus as well as the temporo-parietal area is not only involved in belief based perspective taking during ToM tasks (Aichhorn et al., 2009; Döhnel et al., 2016), but also in directing the attentional focus toward behaviorally relevant objects (Corbetta et al., 2008). Interestingly, the activity increase in the ASD group is not influenced by the congruency of the beliefs.

Although the autistic participants executed the belief task as well as the neurotypical participants fMRI results indicate that there are differences between autistic and neurotypical individuals on the neural level. This result contradicts the study of Dufour et al. (2013) that investigated also belief reasoning but found no group differences between individuals with ASD and NT individuals. But in contrast to our study, they did not differentiate between the computation and question or response phase. We found differences between ASD and NT individuals only in the question phase, in which participants had to refer to a previously encoded observable story line (the movement of the ball and the boxes) in order to infer an unobservable belief. It might be that ASD individuals compared to NT individuals have to recruit a broader network of brain areas in order to respond adequately to questions concerning beliefs. Additionally, also the conjunction analysis of common functional activity associated with the processing of incongruent beliefs in the story and the question phase may point to subtle differences in the neural processing between ASD and NT individuals. By using a more liberal threshold the ASD group showed increased activity in the pMPFC in both phases during processing incompatible beliefs. Possibly, these differences in the neural processing play no role in a belief task executed under scientific, and for the participants' predictable conditions. But they may have an influence on the deficits of ASD individuals to infer mental states in ongoing social situations in everyday life.

It is important to note that our sample size was rather small which may reduce the chance to detect neural differences between ASD and NT individuals during the processing of different beliefs and may increase the risk that results in neuroimaging studies are not reproducible. However, the revealed activity of the whole group during story processing was very similar to our previous study (Schuwerk et al., 2014). And also Dufour et al. (2013) found no differences in brain activation during a false belief task in adults with and without autism. Additionally, the behavioral data showed that the ASD group performed equally well on the unexpected transfer task as the NT group. Therefore, for future research it could be very interesting to investigate how individuals with ASD compensate their mentalizing deficits in such tasks.

## Summary

The current study was the first one that used the classic unexpected transfer task in order to investigate the neural correlates of false belief reasoning in high-functioning and Asperger adults. Our adapted version of the task makes it possible to separate a phase in which diverging beliefs have to be computed from a phase in which participants have to respond either due to a diverging belief or to a belief that corresponds with their own belief and reality. According to former studies we found no differences between our ASD participants and our NT participants on a behavioral level. On the neural level both groups showed similar activation patterns during the processing of the story, but during the response phase ASD individuals showed increased activity in fontal and temporoparietal areas. Possibly, these subtle processing differences may contribute to the fact that adult ASD individuals are able to master explicit false belief tasks (Ponnet et al., 2004; Scheeren et al., 2013) but fail to apply their strategies during naturalistic social interactions.

## Author contributions

MoS: substantial contributions to the conception and design of the work; the acquisition, analysis, and interpretation of data for the work; writing the manuscript; KD, IJ, and VN: substantial contributions to the conception and design of the study; the acquisition of the patients and the analysis of the data; TS, LB, MaS, and RR: substantial contributions to the conception and design of the work; the acquisition and Interpretation of the data and the revising the manuscript. MoS, KD, LB, MaS, VN, TS, and RR: final approval of the manuscript to be published; Agreement to be accountable for all aspects of the work.

### Conflict of interest statement

The authors declare that the research was conducted in the absence of any commercial or financial relationships that could be construed as a potential conflict of interest.
